# Prevalence and Genetic Diversity of Torque teno felis virus (FcTTV) in Domestic Cats from Kazakhstan

**DOI:** 10.3390/v17091265

**Published:** 2025-09-19

**Authors:** Gulzhan Yessembekova, Bolat Abdigulov, Alexandr Shevtsov, Asylulan Amirgazin, Sarsenbay Abdrakhmanov, Elena Shevtsova, Symbat Bolysbekkyzy, Salima Baduanova, Alexandr Shustov

**Affiliations:** 1 Faculty of Veterinary and Animal Husbandry Technology, S. Seifullin Kazakh Agrotechnical University, Astana 010000, Kazakhstan; s_abdrakhmanov@mail.ru (S.A.); simbat.bolisbekkizi@mail.ru (S.B.); 2 National Center for Biotechnology, Astana 010000, Kazakhstan; ncbshevtsov@gmail.com (A.S.); asylulan0894@gmail.com (A.A.); elenashe@mail.ru (E.S.); s.baduanova@biocenter.kz (S.B.); shustov@biocenter.kz (A.S.)

**Keywords:** Torque teno felis virus (FcTTV), *Anelloviridae*, domestic cats, genetic diversity, phylogenetic analysis

## Abstract

Anelloviruses have a broad mammalian host range, including Torque teno felis virus (FcTTV), a felid-associated member that remains undercharacterized. This is the first comprehensive study of FcTTV in domestic cats in Central Asia. We analyzed blood samples from 206 domestic cats from the large city of Astana, Kazakhstan, collected in 2023–2024. Using nested PCR we identified 63 FcTTV-positive samples (30.6% prevalence), and the sequences were compared to global reference strains. Potential demographic associations (sex and age) were assessed. The study revealed an overall FcTTV prevalence of 30.6%. Infection rates showed no significant sex-related differences: ages varied 4–168 months. ORF1 sequencing revealed multiple FcTTV variants in 27% of samples, with no demographic links. Phylogenetic analysis revealed distinct patterns at both nucleotide and amino acid levels: 3 groups of nucleotide sequences (max divergence 21.68%; intra-cluster 5.15–6.8%), and 3 clusters of amino acid sequences (max divergence 16.81%; intra-cluster 2.82–6.68%). Deletions were found in ORF1 in some variants. Global phylogeny aligned clusters with Asian/European strains (90–98% identity), confirming FcTTV1 affiliation and inter-regional transmission. Our study of FcTTV in Kazakhstan reveals moderate virus prevalence with considerable genetic diversity across viral strains and frequent co-infections with multiple variants.

## 1. Introduction

The *Anelloviridae* family is represented by small, non-enveloped viruses, which harbor negative-sense single-stranded DNA genomes of fairly compact size (1.6–3.9 kilobases). Viruses in this family show broad vertebrate host ranges, including humans; nevertheless, most species exhibit marked host specificity. Anelloviruses dominate the human blood virome, comprising over 70% of all viral sequences detected in healthy individuals [1]. This strikingly high prevalence establishes *Anelloviridae* as the dominant human virome [2]. The archetypal Torque teno virus (TTV) was first discovered in 1997 in a patient with post-transfusion hepatitis of unknown etiology [3]. Since its discovery, TTV taxonomy has undergone multiple revisions and expansions. The advent of high-throughput sequencing (NGS) and metagenomic approaches has particularly accelerated this process [4,5,6]. The Anelloviridae family (ICTV 2024) contains 34 genera and 173 species [7]. The species-level classification is based on the level of nucleotide homology in the ORF1 gene: strains with an identity below 69% are considered to belong to different species [8,9].

Despite the high prevalence of anelloviruses in humans and other mammals, the role of these viruses as causative agents of diseases is not yet clear. During the first decade after the discovery of TTV, a significant number of studies focused on identifying associations with diseases and substantiating TTV as a potential etiological agent [10], including in viral hepatitis [11,12], autoimmune diseases [13], and multiple sclerosis [14]. On the other side, the near-universal prevalence of anelloviruses in humans (approaching 100% in adults) has prompted the ‘beneficial virome’ hypothesis, which postulates that these viruses may be beneficial by stimulating the immune system in early childhood. Consequently, TTV has emerged as a biomarker of the immune status, with a clinical utility in monitoring transplant recipients. The TTV viral load provides an assessment of the immune system state upon organ transplant [15,16].

Although domestic cats are the most prevalent companion animals and live in close contact with humans, data on anellovirus circulation in cats remain limited [17,18,19,20]. Better characterization of these viruses in cats is essential to resolve Anelloviridae diversity, host specificity, and their possible zoonotic significance. Comprehensive FcTTV monitoring helps to evaluate the clinical significance of this virus, including risks to pet owners.

Here we present the first study of FcTTV prevalence and genetic diversity in Kazakhstan’s domestic cats.

## 2. Materials and Methods

### 2.1. Samples and Data Collection

This study analyzed whole blood samples collected from 206 domestic cats examined at two veterinary clinics in Astana between September 2023 and December 2024. Demographic data included age (median: 60 months; range: 4–168 months; *n* = 101) and sex (53.6% female, 46.4% male; *n* = 166), with most animals being mixed-breed (*n* = 196) and a subset diagnosed with infectious diseases (*n* = 16: panleukopenia, *n* = 10; rhinotracheitis, *n* = 1; peritonitis, *n* = 5). Samples were collected in K2EDTA vacuum tubes, stored at +4–8 °C during transport (≤24 h), and processed following ethical approval from the S. Seifullin Kazakh Agrotechnical University (protocol code 1, dated 9 January 2022).

### 2.2. DNA Isolation

DNA isolation was performed using the method described by Kanai et al. [21], with minor modifications. Blood samples (including clotted and pre-frozen; volume 200 µL) were transferred to sterile 1.5 mL microcentrifuge tubes containing 250 µL of lysis buffer (360 µg/mL of proteinase K, 150 mM NaCl, 50 mM EDTA, 2% SDS). Incubation was carried out at 60 °C for 3 h with periodic stirring on a vortex. After lysis, 150 µL of 6 M NaCl and 600 µL of chloroform-isoamyl alcohol (in a 24:1 ratio) were added to the tubes, then thoroughly mixed. Centrifugation was performed at 1677× *g* for 5 min for phase separation. The upper phase containing nucleic acids was transferred to a new tube with 800 µL of 90% ethanol for DNA precipitation. The samples were centrifuged at the same parameters, after which the precipitate was washed with 70% ethanol. The resulting precipitate was dried at room temperature and resuspended in 100 µL of TE buffer (10 mM Tris-HCl, 1 mM EDTA, pH 8.0).

### 2.3. PCR Testing

Detection of FcTTV was performed using nested polymerase chain reaction (PCR) with primers described by Veronika Jarošová and co-authors [18]. Primers P1a (5′-AGTAAGTACACTGACGAATGGCTGA-3′) and P2a (5′-CAGTTACCACAGTTCGAGGTCGT-3′) were used for the first round of amplification, and P3a (5′-ACTGGTGACAGGACGCGA-3′) and P4a (5′-TGCGGAGACAAGTTGCTTCC-3′) were used for the second round. PCR amplification was performed in 25 µL reaction mixtures containing 10 pmol of each primer, 10 mM Tris-HCl (pH 8.8 at 25 °C), 50 mM KCl, 0.08% (*v*/*v*) Nonidet P40, 2.5 mM MgCl_2_, 200 µM of each dNTP, 1.5 U Hot Start Taq DNA polymerase (BiolabMix, Novosibirsk, Russia), and either 5 µL of template DNA (first round) or 3 µL of PCR product (second round). Reactions were carried out in a Mastercycler ProS thermal cycler (Eppendorf, Hamburg, Germany) with an initial denaturation at 95 °C for 5 min, followed by 32 cycles of 95 °C for 30 s, 57 °C for 30 s, and 72 °C for 50 s, and a final extension at 72 °C for 5 min. Amplification products were analyzed by electrophoresis on 1.5% agarose gels stained with ethidium bromide and visualized under UV light.

### 2.4. Sequencing ORF1

Complete ORF1 was amplified using nested PCR using primers P1 (5′-TCGCACGTCCTGTCACCAGT-3′) and P2 (5′-GGAAGCAACTTGTCTCCGCA-3′) for the first round of amplification, and primers P3 (5′-TCAGCCATTCGTCAGTGTACTTACT-3′) and P2 (5′- GGAAGCAACTTGTCTCCGCA-3′) [18]. PCR amplification was performed in 25 µL reactions containing 12.5 µL of 2× BioMaster HS-Taq PCR-Sp (BiolabMix, Novosibirsk, Russia), 1 µL (10 pmol/µL) of each round-specific primer, either 5 µL of template DNA (first round) or 3 µL of PCR product (second round), and nuclease-free water to adjust the final volume. Thermal cycling was conducted on a Mastercycler ProS (Eppendorf, Hamburg, Germany) with an initial denaturation at 95 °C for 5 min, followed by 32 cycles of 95 °C for 30 s, 63 °C (first round) or 61 °C (second round) for 20 s, and 72 °C for 3 min, with a final extension at 72 °C for 15 min. The resulting ~1750 bp amplicons were purified using AMPure XP beads (Beckman Coulter, Beverly, MA, USA) at a 1:1 ratio, followed by library preparation with the Illumina DNA Prep kit (Illumina, San Diego, CA, USA) and sequencing on the MiSeq platform using a 300-cycle reagent kit.

Raw sequence data were first quality-checked using FastQC v0.12.1 (https://github.com/s-andrews/FastQC, accessed on 8 September 2025), followed by read trimming and quality filtering with Seqtk v1.3 (https://github.com/lh3/seqtk, accessed on 8 September 2025) and Sickle v1.33 (https://github.com/najoshi/sickle, accessed on 8 September 2025) to remove adapters and low-quality bases. High-quality reads were assembled de novo using the MEGAHIT v1.2.9 [22] metagenomic data assembler. The assembler was chosen due to its ability to generate long and accurate contigs from short reads, especially those obtained from co-infected samples [23,24]. Subsequently, the obtained contigs were analyzed to identify and retrieve complete FcTTV ORF1 sequences.

### 2.5. Phylogenetic Analysis

Phylogenetic relationships were inferred by maximum likelihood (ML) under the Hasegawa–Kishino–Yano (HKY) substitution model [25], which was identified as the best-fit model using the “Find Best DNA/Protein Models” function in MEGA X [26]. Bootstrap support values are based on 1000 replicates. Evolutionary rate variation among sites was modeled using a discrete Gamma distribution with 5 categories (+G parameter).

## 3. Results

### 3.1. Prevalence and Genetic Diversity of FcTTV in Domestic Cats

FcTTV DNA was detected in 30.6% (63/206) of sampled cats. Among PCR-positive animals with recorded sex (*n* = 58), the distribution was nearly equal between females (48.3%, 28/58) and males (51.7%, 30/58), with no significant sex-based difference (χ^2^ = 0.069, *p* = 0.793). For age-stratified analysis (*n* = 43), infected cats ranged from 4 to 168 months old (median: 72 months). Concurrent infections were rare, with only three cases identified: two feline infectious peritonitis (FIP) diagnoses and one parvovirus enteritis.

Despite positive results in short-amplicon diagnostic PCR, amplification of the full-length ORF1 failed in 26/63 FcTTV-positive samples (41.3%), possibly due to low target copy number, template DNA degradation, and/or suboptimal long-amplicon PCR efficiency. Full-length ORF1 sequencing was successful in 37/63 FcTTV-positive samples (58.7%), revealing coinfection with two distinct viral variants in 10 samples (27%). No correlations were found between variant coinfection and host age or sex.

**Figure 1:**
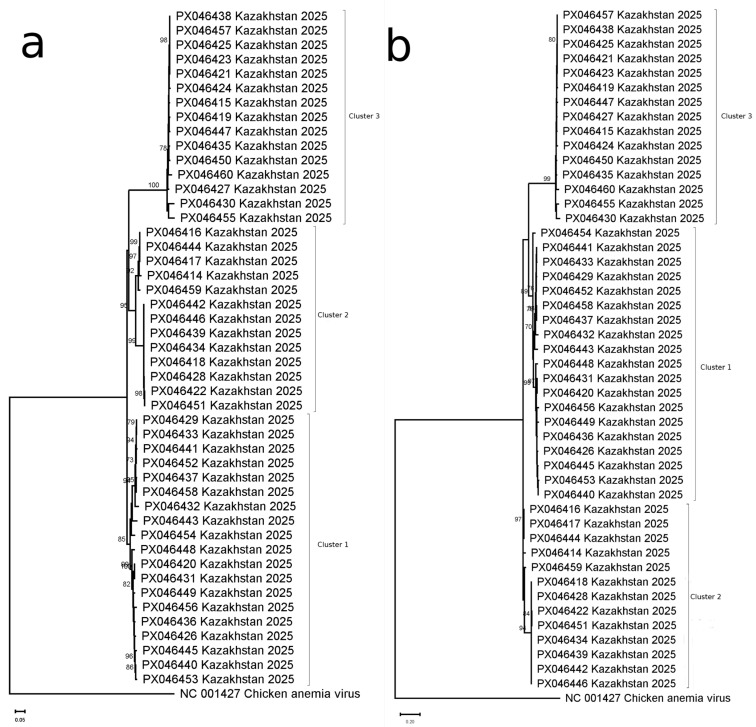
Phylogenetic analysis of FcTTV variants. (**a**) Nucleotide-based tree constructed from 47 complete ORF1 sequences (derived from 37 samples, including 10 with confirmed co-infections). (**b**) Corresponding amino acid-based phylogeny from the same sample set. Both trees demonstrate clustering patterns of viral variants, with co-infected cases marked.

Phylogenetic analysis of nucleotide sequences (*n* = 47) identified three major clusters comprising 35 genetic variants (Figure 1a, Table S1), with maximum inter-cluster divergence of 18.0%. Cluster-specific divergence levels were 5.9% (Cluster 1), 6.4% (Cluster 2), and 4.9% (Cluster 3), with ORF1 lengths of 1311 bp (Clusters 1–2) and 1302 bp (Cluster 3).

Amino acid analysis confirmed this virus genotypic structure (Figure 1b), resolving 33 variants with maximum divergence of 28.9% (Cluster 1: 8.5%; Cluster 2: 10%; Cluster 3: 6.4%). Notably, Cluster 3 variants lacked three amino acids present in the reference strain from Genbank (AB076003) (deletions were found in positions 44, 275, and 276), which was verified by read mapping on ORF1 assembly.

### 3.2. FcTTV Isolates in Kazakhstan as Compared to Global Diversity

**Figure 2:**
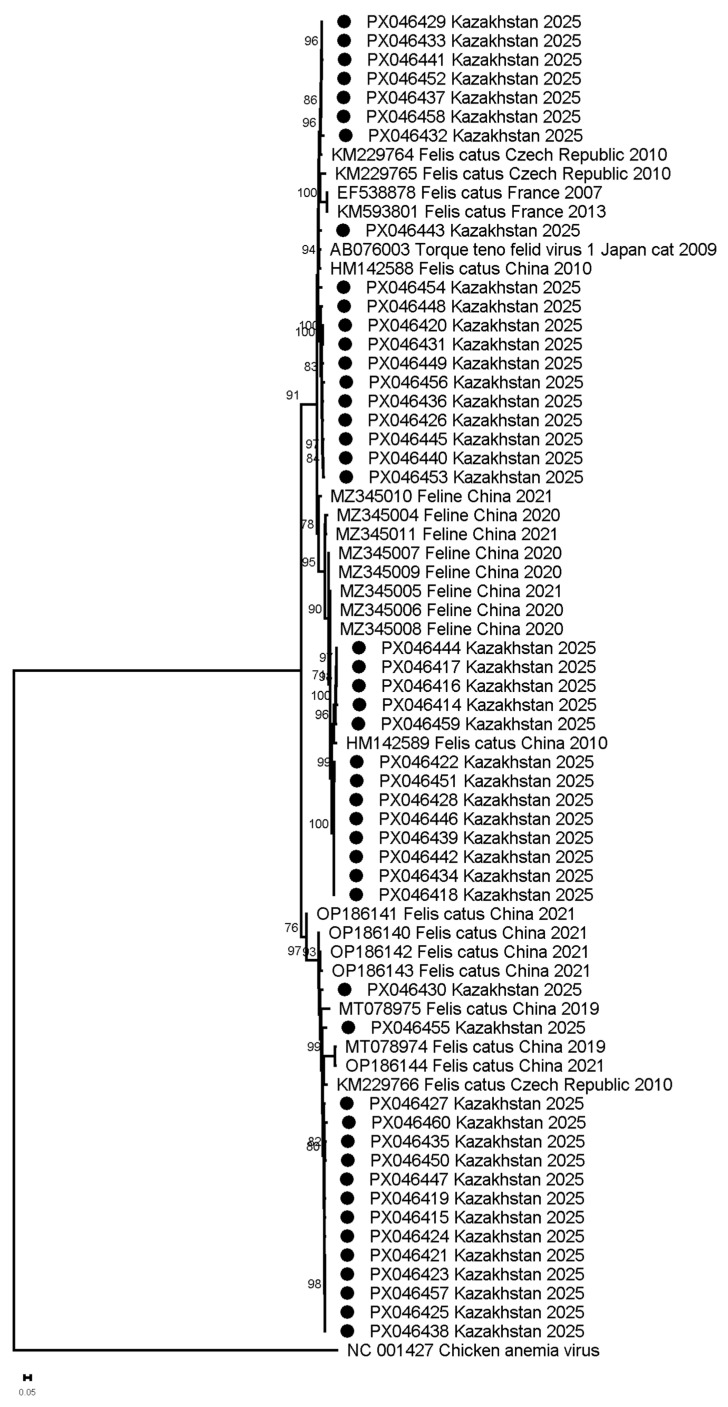
Maximum-likelihood phylogeny of FcTTV ORF1 nucleotide sequences. (Isolates from Kazakhstan indicated by circles; reference sequences from GenBank are labeled with accession numbers. The tree was constructed using HKY + G model (1000 bootstrap replicates) with scale bar showing substitutions/site. Reference strains include AB076003 (prototype) and representative Asian/European variants.

FcTTV sequences from Astana showed maximum divergence 17.09% from reference AB076003 and thus were classified as genotype 1 (FcTTV1). At the same time, isolates from Kazakhstan show grouping into three clusters with distinct geographic associations: Cluster 1 strains (93.5–98% identity) aligned with AB076003 and variants from China (HM142588) and the Czech Republic (KM229764, KM229765); Cluster 2 (94.1–95.7% identity) included a 2010 Chinese strain (HM142589); and Cluster 3 (91.2–97.8% identity) grouped with six Chinese and one Czech variant. In contrast, French sequences formed a separate branch with lower identity (83–94.5%), suggesting regional divergence alongside historical cross-continental transmission (Figure 2, Table S1).

## 4. Discussion

The current study is the first of its kind on the prevalence and genetic diversity of TTV among domestic cats in Kazakhstan. Recently, the number of studies on FcTTV in domestic cats has significantly declined due to a lack of targeted research. The scientific focus has shifted toward anellovirus species in other host animals [27,28,29]. However, the topic remains under-researched, as previous studies have been geographically limited to the Czech Republic and Shanghai [17,18]. Our study, which revealed a prevalence of 30.6% and a genetic divergence of up to 21.68% in ORF1, contributes to this area by highlighting regional specificities in Kazakhstan and confirming the need for monitoring for veterinary epidemiology with the expansion of the studied animal species and sampling range.

A study of the carriage of Torque teno felis virus (FcTTV) in the Astana cat population revealed a prevalence of 30.6% (63/206 samples), which is consistent with previously obtained prevalence values among domestic cats in Shanghai (36.67%) and the Czech Republic (33.63%) [17,18]. FcTTV infection rates observed in domestic cats appear moderate when contrasted with reports of near-universal (up to 100%) anellovirus prevalence in certain human and ani-mal populations [30,31]. TTV prevalence exhibits marked interspecies variation, with particularly wide ranges observed in domestic pigs (TTSuV: 17–100% across and within countries) [32,33,34], whereas in domestic dogs the virus is present in 7–38% [35]. However, keeping pets in confined and stressful conditions often results in decreased immunity, which may explain the high rates of anellovirus infection. In wild animals, TTV infection also varies, with wild pigs infected worldwide ranging from 32% to 66% [36], 87% of wild forest mice in the UK and 100% of forest martens are infected with TTV [37,38]. In stool samples from mouse-like rodents from 6 provinces of China, TTV was detected in 29.1% of samples. [39]. The consistent TTV prevalence among domestic cats across three countries (30.6–36.67%) presents intriguing epidemiological stability compared to other species. However, current infection rates may underestimate true prevalence due to: (1) non-standardized detection methods, and (2) high genetic diversity enabling variant escape from conventional PCR assays—a known challenge in anellovirus surveillance [10]. Our diversity estimates also should be interpreted with caution. Targeted PCR can underrepresent phylogenetically distant lineages, co-infections, and rare variants due to primer bias, template integrity requirements, and limitations of long-amplicon PCR. Unbiased virome/metagenomic sequencing is needed to capture the full spectrum of circulating anelloviruses.

The balanced sex distribution among infected cats (females: 48.3%, males: 51.7%; *p* = 0.793) aligns with established anellovirus epidemiology, where host sex typically shows no association with infection status [40]. Infected cats showed a higher median age (72 months vs. 60 months in the general population), consistent with the chronic infection pattern observed for TTV in humans and other mammals, where prevalence correlates positively with host age [41,42,43].

We detected coinfection with multiple FcTTV1 variants in 27% (10/37) of sequenced samples, revealing substantial mixed infections in Astana’s cat population. This frequency aligns with global anellovirus patterns, where coinfection rates often surpass single-variant infections, facilitating viral diversification through recombination and enhanced replication dynamics [36,44]. Similar evidence of recombination, both within and between different groups of anelloviruses, has been observed in wild cats, supporting a long history of host–virus coevolution [45]. In pigs (TTSuV), coinfections are even more common than infections with a single variant [44], and mixed infections with different genotypes are common in humans [46].

Phylogenetic analysis of ORF1 sequences identified three distinct FcTTV clusters among Astana isolates, exhibiting diversity but consistent with a genetic variation within a single species [9]. Phylogenetic analysis incorporating multiple reference strains from GenBank revealed distinct geographic patterns: two clusters contained both Asian (Kazakhstan, China) and European (Czech Republic) variants (90–98% identity), while the third cluster comprised exclusively Asian strains (>95% identity to Chinese isolates, 2010–2023). This phylogeographic structure suggests inter-regional transmission possibly linked to international cat trade, and localized evolution of the Asian cluster [47].

## 5. Conclusions

This first comprehensive study of Torque teno felis virus (FcTTV) in domestic cats in Central Eurasia reveals high prevalence (30.6%), significant genetic diversity (up to 21.68% ORF1 divergence), and frequent co-infections (27% of sequenced samples), which complements the epidemiological picture of anellovirus infection. Phylogenetic evidence of shared FcTTV genotypes between Asian and European strains suggests global prevalence, supposedly the result of the international trade of cat breeds.

## Data Availability

All data from this project is publicly available NCBI, GenBank Ac#: PX046414-PX046460.

## Supplementary Materials

The following supporting information can be downloaded at: https://www.mdpi.com/article/10.3390/v17091265/s1, Table S1: Nucleotide sequence divergence in the ORF1 region between FcTTV1 strains detected in Astana and globally circulating variants; Table S2: Level of divergence in the amino acid sequence of ORF1 among FcTTV strains circulating in Astana.
